# HPV and Other Microbiota; Who’s Good and Who’s Bad: Effects of the Microbial Environment on the Development of Cervical Cancer—A Non-Systematic Review

**DOI:** 10.3390/cells10030714

**Published:** 2021-03-23

**Authors:** Matthias Läsche, Horst Urban, Julia Gallwas, Carsten Gründker

**Affiliations:** Department of Gynaecology and Obstetrics, University Medicine Göttingen, 37075 Göttingen, Germany; mlaesch@gwdg.de (M.L.); horst.urban@med.uni-goettingen.de (H.U.); julia.gallwas@med.uni-goettingen.de (J.G.)

**Keywords:** cervical cancer, metabolism, microbiota, HPV

## Abstract

Cervical cancer is responsible for around 5% of all human cancers worldwide. It develops almost exclusively from an unsolved, persistent infection of the squamocolumnar transformation zone between the endo- and ecto-cervix with various high-risk (HR) human papillomaviruses (HPVs). The decisive turning point on the way to persistent HPV infection and malignant transformation is an immune system weakened by pathobionts and oxidative stress and an injury to the cervical mucosa, often caused by sexual activities. Through these injury and healing processes, HPV viruses, hijacking activated keratinocytes, move into the basal layers of the cervical epithelium and then continue their development towards the distal prickle cell layer (*Stratum spinosum*). The microbial microenvironment of the cervical tissue determines the tissue homeostasis and the integrity of the protective mucous layer through the maintenance of a healthy immune and metabolic signalling. Pathological microorganisms and the resulting dysbiosis disturb this signalling. Thus, pathological inflammatory reactions occur, which manifest the HPV infection. About 90% of all women contract an HPV infection in the course of their lives. In about 10% of cases, the virus persists and cervical intra-epithelial neoplasia (CIN) develops. Approximately 1% of women with a high-risk HPV infection incur a cervical carcinoma after 10 to 20 years. In this non-systematic review article, we summarise how the sexually and microbial mediated pathogenesis of the cervix proceeds through aberrant immune and metabolism signalling via CIN to cervical carcinoma. We show how both the virus and the cancer benefit from the same changes in the immune and metabolic environment.

## 1. Introduction

The microbiota is the community of microorganisms in a specific environment. It determines the metabolism and the immune environment of the host tissue through its microcosm and its own demands on metabolism and immune signalling. It has a decisive influence on homeostasis and the health of the organism. In the microbiome of the female lower reproductive tract (vagina and cervix), especially in the part of the cervix reviewed here, the microenvironment of the mucous membranes is colonised by a mixture of microorganisms. These mostly belong to the lactobacilli family [1]. The upper part of the female reproductive tract (uterus, fallopian tubes and ovaries) is either germ-free or populated with a low number of microorganisms in a species-rich manner. If the composition of the genital microbiota changes in such a way that the conditions in the metabolism and immune signalling of the host change due to the displacement of probiotic and settlement of pathological microorganisms, this is referred to as genital dysbiosis. Dysbiosis can also be found on other mucosal sites of the human body. It can initiate the characteristic features of cancer, such as altered immune and metabolic signalling, chronic inflammation, and associated tissue damage. In addition, changes in cell proliferation and cell cycle, aberrations in the stability of the genome as well as epigenetic changes are promoted. Dysbiosis also causes formation of new blood vessels and an aberrant metabolism [2,3]. All of these changes, if they take place over a long period and are not corrected, can lead to malignant changes in the tissue. The latest studies [3] have led to the view that microorganisms and—in the special case of cervical cancer—especially the human papillomaviruses (HPVs) play a decisive role in the development of gynaecological cancer. Nevertheless, the cancer therapies themselves also influence the composition of the microbiome of various mucosal sites and thereby cause serious side effects, which can be alleviated by taking probiotic foods or implanting a probiotic microbiota [4,5,6,7]. For this reason, in this review article, we examine the processes involved in the developing malignancy of cervical cancer with a special focus on the metabolic crosstalk between the developing tumour and its microbial and immune microenvironment.

## 2. The Role of the Microbiota in Carcinogenesis

The female reproductive tract (FRT) can be divided into the upper and lower tracts. The upper tract includes the uterus, fallopian tubes, and ovaries; the lower tract the vagina, cervix, and vulva. The cervix, in turn, can be divided into two zones: (1) the endo-cervix or cervical canal leading into the uterus and (2) the ecto-cervix. At the border between both parts lies the transformation zone (TZ). The cervical canal is narrow and with its cervical mucus, which is produced by the mucus-forming glandular tissue, ensures that no germs and, in the sterile phase of the menstrual cycle, no sperm can rise into it. During pregnancy, it protects the developing life from rising germs by means of a tough plug of mucus. The ecto-cervix forms a stratified squamous epithelium, in contrast to the columnar endo-cervical epithelium. When malignant transformation of the cervix occurs, it mainly takes place in the squamocolumnar TZ [8,9]. Malignant changes may be preferred in this region because the human papillomaviruses, which are responsible for 99.7% of cervical cancer cases [10], infect the basal cells of the epithelium and damage the overlying cell layers, basal cells that—in the TZ—are more easily exposed due to the thinner layer thickness of the overlying cell layers. For its development, the papillomavirus needs the differentiation ability of the basal cells, which is only given in the TZ [11,12]. In addition, hormones such as oestrogen and progesterone not only support the processes of transformation of the cervix during the menstrual cycle and pregnancy [13]; they also promote HPV infection [14] and carcinogenesis [15]. Analogous to this, the same preference is found for the squamocolumnar TZ, similar to the cervical TZ, in the carcinogenesis of anal cancer [16,17]. We will report on the detailed processes during the development of the HPV virus infection and the resulting malignant changes in the cervix in the later part of the review.

With around 570,000 cases and 311,000 deaths in 2018, cervical cancer is the fourth most common cancer worldwide and the most common malignant transformation caused by HPV infection [18]. Furthermore, there are differences in the incidence of the disease among women of different races and ethnic groups, but also depending on their socio-economic origin and access to cervical screening programs [19,20]. These are determined not least by a different composition of the genital microbiota [1,21,22,23], but also by different sexual behaviours, environmental factors and a different genetic and hormonal disposition. There are a number of meta-analyses from an epidemiological point of view that examine the connections and relationships between the host, its own microbiome and the human papillomavirus in the carcinogenesis of the cervix. However, since these will go beyond the scope of this review article, we would like to refer to the review article by Paweł Łaniewski et al. [3].

The community of symbiotic and pathogenic microorganisms and their metagenome (totality of genomic information) determine the tissue homeostasis and the pathogenesis of certain diseases, including cancer [3]. The microbial community that populates the human body consists of bacteria, archaebacteria, fungi, protists, and viruses [24]; and their habitat, along with their genomes and microenvironment, is called the microbiome [25]. It is believed that the number of bacterial microorganisms that colonise the human body is roughly as large as that of the human cells themselves [26]. The bacterial community probably has a metagenome 100 times larger than that of humans [27].

Even if the term “pathobiont” as a symbiont that can trigger pathological processes is incorrect and does not represent the true occurrences in the microenvironment of the diseases [28], we have stuck to this term in order to keep our slightly provocative title for the sake of simplicity and with a view to understanding the whole. Of course, the microbiome is more complex and not black and white and yet it is important to understand which microorganisms in which context could have a pathological or health-promoting effect.

One study showed that changes in the microbiome and metabolome occurred in the early stages of colon cancer development. On the one hand, the relative frequency of *Fusobacterium nucleatum* as well as *Atopium parvulum* and *Actinomyces odontolyticus* increased towards the advanced stages of colon cancer, respectively in multiple polyploid adenomas and/or intramucosal carcinomas. On the other hand, there were changes in the metabolite concentrations, such as increased levels of branched-chain amino acids and phenylalanines in intramucosal carcinomas, and respectively, increased bile acids such as deoxycholate in multiple polyploid adenomas and/or intramucosal carcinomas [29].

In another study [30], three different metabolome clusters were identified that differed in their composition and were related to dysbiotic inflammation-induced cervical carcinogenesis, the pH of the vagina and the presence of HPV infection. Increased levels of 3-hydroxybutyrate, eicosenoate, and oleate/vaccenate indicated disease progression, while sphingolipids, plasmalogens, and linoleate were indicators of inflammation. The non-*Lactobacillus* dominant population altered the metabolism of amino acids and nucleotides. The *Lactobacillus* frequency was related to increased adenosine and cytosine levels and reduced inflammation parameters, the non-*Lactobacillus* frequency, via the glycochenodeoxycholate (GCDC) and carnitine metabolism, on the other hand, was related to the inflammation.

Another study [31] compared three different Community State Type (CST) groups of the vaginal microbiota. The group CST I stood for the healthy group dominated by *Lactobacillus crispatus*. The *Lactobacillus iners* CST III group showed higher levels of biogenic amines, phospholipid and glycogen derivatives in HPV-positive women than in HPV-negative women. The low-*Lactobacillus* group CST IV, which stands for molecular bacterial vaginosis, had lower levels of glutathione, glycogen, and phospholipid derivatives than in HPV-negative women. Based on the relationship between HPV infection and glutathione levels, the authors conclude that antioxidant therapy could be used in the future.

Others analysed the metabolome of 19 women from Puerto Rico using a model-based integration of metabolite observations and species abundance 2 (MIMOSA2) approach and found a connection between the three metabolites excreted in the urine, threonine, proline, acetate (HPV^+^) and their four corresponding enzymes, threonyl-tRNA synthetase, prolyl aminopeptidase, prolyl-tRNA synthetase, and acyl phosphatase. An increased succinate excretion, on the other hand, stood for the healthy, HPV-negative woman. HPV infection increased the diversity of microbial species and their ability to produce or use certain metabolites. This resulted in an increased incidence of *Lactobacillus* sp. and a decreased incidence of *Lactobacillus iners* and *Shuttleworthia*, which affected bacterial homeostasis [32].

It has been known for some time that specific viruses and bacteria increase risk of incidence for specific cancers. An example of a bacterium that has even been described as a carcinogen [33] is *Helicobacter pylori*, which colonises the gastric mucous membrane and can trigger carcinogenesis through chronic inflammation [34]. Gallbladder cancer is probably associated with chronic infection by *Salmonella enterica* serovar Typhi or Paratyphi [35] and specific lymphomas with *Borrelia burgdorferi* or *Chlamydia psittaci* [36,37]. However, seven different viruses are also considered to be carcinogenic [38], such as (1) the Epstein-Barr virus (EBV), associated with the development of Burkitt, Hodgkin, and Non-Hodgkin lymphoma and nasopharyngeal cancer [39], (2) the hepatitis B virus (HBV) and (3) the hepatitis C virus (HCV), both responsible for hepatocellular cancer [40,41], (4) the Kaposi’s sarcoma herpes virus (KSHV), responsible for Kaposi’s sarcoma and primary effusion lymphoma [42,43], (5) the Merkel cell polyomavirus (MCPyV or MCV), which initiates Merkel cell carcinoma [44], (6) the human T-lymphotropic virus 1 (HTLV-1), responsible for adult T-cell lymphoma [45], and (7) the high-risk (HR) types of HPV, 16, 18, 31, 33, 35, 39, 45, 51, 52, 56, 58, and 59, which are decisive for the development of the cervical, vaginal, oropharyngeal, and anal cancer [46,47,48], with HPV 16, 18 and 31 found especially in cervical cancer [49,50]. Bacteria produce special toxins that change the host DNA and thus damage it [2,51]; or the virus DNA itself, for example in the case of HPVs, integrates into the human genome and thus acts as an oncogene [52,53].

The effects of probiotic microorganisms on tissue health or possible disease progression through dysbiotic bacteria are largely determined by the integrity of the mucosal epithelium [2,51,54,55]. Important protective properties are ascribed to the lactobacilli and their main excretion product, protonated lactate, which improve the barrier function of the mucous membrane epithelium of the cervix and protect their microenvironment against invading pathogenic microorganisms due to the acidification to a pH value below 4.5 [3,56]. Dysbiotic bacteria of the female genitals, mostly a diverse mixture of anaerobic bacteria such as *Anaerococcus*, *Atopium*, *Fusobacterium*, *Gardnerella*, *Gemella*, *Parvimonas*, *Peptostreptococcus*, *Prevotella*, *Sneathia, Shuttleworthia*, and others, which displace the probiotic bacteria, influence the natural inflammatory reaction (anti-inflammatory cytokines such as the interleukin-1 receptor antagonists (IL-1RA) and antimicrobial peptides) [1,56,57,58] to a chronic aberrant inflammatory reaction [54]. They destroy the epithelial barrier, generate an aberrant regulation of immune signalling, and lead to an increased production of pro-inflammatory cytokines and chemokines, such as interleukin-6 (IL-6), tumour necrosis factor (TNF), and interleukin-8 (IL-8) [2,59]. The increased level of reactive oxygen species (ROS) with a simultaneous increase in the vaginal pH value [23] causes genomic instability, which can lead to genotoxicity, aberrant proliferation, and apoptotic cell death [2,51]. Bacterial enzymes such as sialidase, a neuraminidase, which is able to eliminate the glycosylations in the glycocalyx of the host via the cleavage of the N-acetylneuraminic acid, are able to break down the mucus that protects against a progressive infection in order to finally remove the epithelial barrier to help pathological bacteria to break through [60]. The changed oxygen saturation of the tissue (hypoxia) also promotes angiogenesis via the Janus kinases (JAK) signal converter and activator of transcription (STAT) pathway and the production of vascular endothelial growth factor (VEGF) [2,3]. Overall, the chronic inflammatory reaction, the activation of the core factor ‘kappa light chain enhancer’ of activated B cells (NF-κB), the toll-like receptors (TLRs), and the nucleotide-binding oligomerisation domain-like receptors (NLRs) create a tumour-promoting microenvironment that enables cancer to occupy the innate and adaptive immune system for its own purposes and to create a resistance to cell death [2,51,54,55,60]. Pathogenic bacteria in the intestine are able to promote IL-17–IL-23 signalling via increased levels of IL-6 and TNF and the activation of the STAT-3 pathway [2,59,61]. Other bacteria, such as *Helicobacter pylori*, stimulate the typical characteristics of cancer, such as proliferation, survival, migration, and angiogenesis via the Wnt–ß-catenin signalling pathway [62,63]. *Chlamydia trachomatis*, in turn, is able to carry out the epithelial–mesenchymal transition (EMT) via reduced E-cadherin and increased N-cadherin levels and the expression of SNAIL1, and the associated loss of cell adhesion between the epithelial cells. Together with the blockade of the DNA damage response, this leads to the promotion of tumour development [64].

## 3. Metabolism in the Microenvironment of Cervical HPV Infection and Neoplastic Transformation

**Figure 1:**
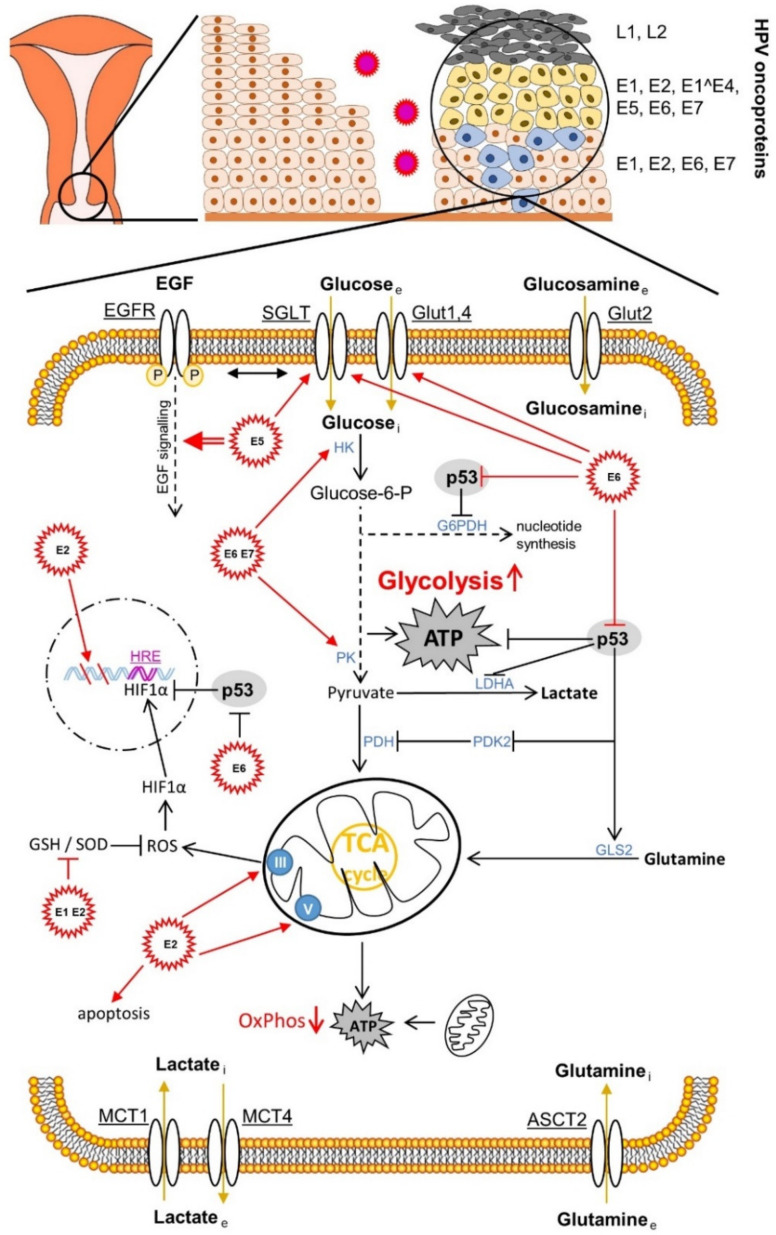
Effects of human papillomavirus (HPV) oncoproteins on cervical cancer cell metabolism. The mechanisms shown are mainly described in Section 4.4. ASCT2, amino acid transporter2; EGF, epidermal growth factor. EGF receptor; G6PDH, glucose-6-phosphat-dehydrogenase; GLS2, glutaminase 2; Glut, glucose transporter; GSH, glutathione; HRE, hypoxia response element; HIF1α, hypoxia-inducible factor 1α; HK, hexokinase; LDHA, lactate dehydrogenase A; MCT, monocarboxylate transporter; OxPhos, oxidative phosphorylation; PDH, glucose-6-phosphat-dehydrogenase; GLS2, glutaminase 2; Glut, glucose transporter; GSH, glutathione; HRE, hypoxia response element; HIF1α, hypoxia-inducible factor 1α; HK, hexokinase; LDHA, lactate dehydrogenase A; MCT, monocarboxylate transporter; OxPhos, oxidative phosphorylation; PDH, pyruvate dehydrogenase; PK, pyruvate kinase; PDK2, pyruvate dehydrogenase kinase isoform 2; ROS, reactive oxygen species; SGLT, sodium glucose transporter; SOD, superoxide dismutase.

The human papillomavirus is associated with about 5% of all human cancer developments worldwide and in the majority of cases (99.7%) with cervical cancer [65]. In addition to the aberrant immune signalling, the metabolism of the host cells is also influenced by the chronic virus infection [66,67,68]. We now want to focus on the metabolism of the cervical microenvironment in the progressive HPV infection. An important feature from which both cancer and the HPV virus could benefit is the altered glucose metabolism [69,70,71,72,73,74,75] (Figure 1).

It is known that the altered glucose metabolism covers the changing need for energy and metabolic building blocks in the course of the changing microenvironment during cancer progression, the dissemination of cancer stem cells, and metastasis [69,72,76,77,78,79].

Otto Warburg postulated aerobic glycolysis, also referred to as the “Warburg effect”, in the early 1920s as a characteristic feature in cancer tissue, in contrast to non-malignant tissue [80,81,82]. He assumed that this was a malfunction due to damaged mitochondria [81]. Today we know that the switch from aerobic cellular respiration to aerobic glycolysis takes place in spite of the sufficient oxygen supply of the cancer tissue for the purpose of faster energy production due to the aberrantly increased proliferation [83], migration [84], and invasion [85]. In addition to the approximately 100 times faster production of energy molecules in form of ATP in glycolysis [86], there is also the need for intermediate metabolites, which branch off mainly from the metabolic pathway of glycolysis, a need caused by the increased speed of cell division and for the required synthesis of cell membranes [87]. The shift in metabolism away from mitochondrial respiration towards lactate production [88] and the resulting acidification of the microenvironment shows immuno-modulatory effects on the host’s immune response (immunosuppression); and the virus and cancer progression ultimately benefit to the same extent through the possibility of evasion of the immune system [89]. Regarding the details of lactate metabolism, however, due to the complexity of the process, we refer to our review article [72]. The synthesis of viral DNA, which is carried out by the viral E1 helicase/ATPase protein—as the only enzymatically active viral protein—during the replication of the HPV genome (processing and splitting of the DNA strands and recruiting the necessary DNA polymerases) also requires a lot of energy. Therefore, the same glycolytic conditions are beneficial to both the virus and cancer [90]. For replication, the virus also needs increased levels of nucleotides, which are delivered via the pentose phosphate pathway (PPP), which splits off from the glycolytic pathway [77]. The glycolytic intermediate glucose-6-phosphate (G6P) is converted by the metabolic enzyme glucose-6-phosphate dehydrogenase (G6PDH), a rate-determining protein in PPP, via ribose-5-phosphate to the nucleotide precursor molecules [75]. During this reaction, redox equivalents that are important for the cell are produced in the reduced form of nicotinamide adenine dinucleotide phosphate (NADPH), which is an antioxidant source for neutralising reactive oxygen species (ROS) and a coenzyme for important reactions in human cells [72,91]. The “Warburg effect” or aerobic glycolysis therefore supports both cancer progression [92] and the replication of the HPV genome in infected cervical cells [90,93,94,95,96].

The mechanisms of the effects of the HPV oncoproteins on the metabolism are described in more detail under point 4.4.

## 4. Tumourigenesis of Cervical Cancer Because of the Special Tissue Anatomy of the Cervix and the Genetic Structure of the HPV Genome

In order to understand why the HPV virus specifically affects the squamocolumnar transition zone (TZ) between the endo- and ecto-cervix and under what conditions a chronic infection leads to a malignant event, one must understand how the TZ is structured and the genome of the virus is organized, as it determines the individual phases of virus replication and the further fate towards cancer progression.

In addition to the special location of the cervix in the woman’s vagina and the associated reaction mechanisms of the cells after injury and healing, when activated keratinocytes carry the virus into the wounded area [13], it is the ability of the border region between the endo-cervical columnar epithelium and the ecto-cervical squamous epithelium to differentiate, which is crucial for virion production [11]. As already mentioned at the beginning, the thin layer thickness of the TZ makes it easier for the virus to infect the two classes of basal cells, the class of basal stem cells and the class of supra-basal transit amplifying (TA) cells [97]. It has not yet been clarified which of the two cell classes gets infected or whether both get infected. The persistence of the HPV infection and thus the likelihood of increased malignant progression of the cervical tissue could be due to the infection of the basal cells and their stem cell character, while a short-term infection would be associated with the infection of the TA cells. However, the virus could also change the cell type more in one direction or the other, so that one time it favours permanent and another time short-term infection, which would suggest a smooth transition of the proliferation potential in the basal cells [66,97].

The genetic structure of the HPV genome in its common organisation determines the precise sequence of replication events of all (more than 200) HPV subtypes [67]. The differences in phenotype between the HPV high-risk (HR) and low-risk (LR) subtypes can be found in the aberrations of the translated amino acid sequence of the common gene segments [98], which are responsible for the encoded functions of the early (Early, E) E1–E8 and the late (Late, L) L1 and L2 genes. According to their functionality, a distinction is made between three groups of genes that are differentially transcribed during the differentiation of the host tissue in coordination with their function. These include the early E6 and E7 gene segments in the first group, which change the functionality of cell cycle regulators. The other early gene segments of the second group, E1, E2, E4, E5, and E8, participate in viral DNA replication, control of transcription, and other later functions that we will describe afterwards. In the initiation phase, E1 and E2 ensure, on the one hand, that a small number (approximately 100) of episomal genome copies are maintained in the undifferentiated basal cells [99], and on the other hand, together with E6 and E7, the replication and amplification of the genomes in the differentiated supra-basal cell layers is ensured [100,101,102,103]. The low expression of viral proteins in the undifferentiated basal cells probably serves the evasion of immune recognition and virus persistence. The L1 and L2 genes of the third group code for the capsid proteins that encase the HPV genomes in the outermost layers of the cervical squamocolumnar TZ. The viral life cycle is finally completed—in a passive manner (i.e., without cell lysis or necrosis)—together with the uppermost epithelial layers that are removed, in order to spread again, mostly promoted by sexual activities [104].

The prerequisite for the initiation of cervical carcinogenesis is the persistence of the HPV infection in the undifferentiated basal cells. For this purpose, the virus must ensure that not only extrachromosomal gene copies are made, but that the virus genome is bound to the host genome, since extrachromosomal copies of the virus DNA would be lost during mitotic cell division. The E2 protein seems to be responsible for this, as it is able to bind to the host chromatin during mitosis; and by binding to the promoter, that regulates the expression levels of E1, E2, E6, and E7, it has the ability to self-regulate even its own gene expression [105,106]. In addition to E2, there exists also its shortened variant E8^E2C, which controls the HPV DNA copy number by inhibiting gene expression, but which, when mutated, is deprived of its functionality [107].

During the transition from the undifferentiated to the differentiated part of the TZ, an increased viral protein biosynthesis only occurs in the direction of the most highly differentiated cell layers. This shift of the increased gene expression into the outer (distal) boundary of the TZ has the strategic advantage that it is shifted to zones of the tissue that are largely beyond immune surveillance. The late L1 and L2 proteins are only expressed in the spinous layer (prickle cell layer, *Stratum spinosum*) [108,109,110].

However, the HPV virus has to overcome one hurdle on the way to the completion of its replication cycle. It must break the cell cycle arrest that is usually present in the differentiating host cells and induce the host tissue to start replication again, since it is dependent on the host’s replication enzymes. The early E7 virus protein, by binding to members of the retinoblastoma (pRb) family, inactivates them, forcing the keratinocytes of the distal epithelial layers back into the cell cycle. This strongly stimulates the transcription of the virus genes E1, E2, E1^E4 (connection of E1 amino acids with the open reading frame of E4), and E5 [111,112,113] and the resulting replication to several thousand DNA copies per cell [114]. The late virus genes E1, E4, and E5 are required during the differentiation of the keratinocytes and the initiation of the late virus functions [115,116,117]. The Ataxia telangiectasia, mutated (ATM)-mediated DNA damage path is activated by E7 and required for replication at so-called replication foci and the subsequent amplification [99]. The expression of the highly immunogenic virus capsid proteins L1 and L2 is delayed until the end of the replication cycle of the viral DNA, in order to achieve the longest possible evasion of the immune response. This happens over several levels, such as the splice donor/acceptor regulation, the change of an early to late poly-adenylation signal (PAS), the transcript stability regulation, and the incorporation of rare codons [118]. Finally, the ready-packaged virions, without having provoked an immune-relevant inflammatory reaction, are released together with the flaked outermost epithelial layers in order to restart their development process [108].

### 4.1. The Maintenance of HPV Immune Evasion and Persistence of the HPV Infection Are Determined by Viral Proteins

Both the innate and adaptive immune systems are cooperatively suppressed by several mechanisms that result from the effects of the early HR E5, E6, and E7 virus proteins and the late L2 protein on the host’s immune responses. The HPV infection usually leads to a corresponding immune response, which in the best case eliminates the virus via regression of the lesions [119]. In case of a persistent infection and the negative effects of an accompanying pathological microbiota [2], the immune system will no longer be able to completely control and eliminate the viral disease [120,121].

**Figure 2:**
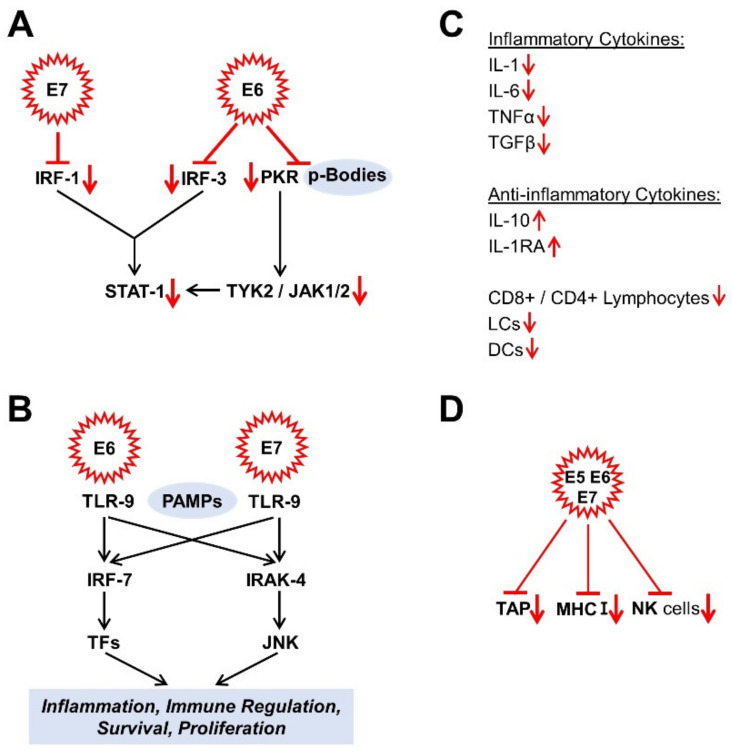
Effects of HPV oncoproteins on immune response. (**A**) signal converter and transcription activator 1 (STAT-1) -signalling; (**B**) Toll-like receptor 9 (TLR9) signalling; (**C**) Cytokines; (**D**) Immune response. The mechanisms shown are mainly described in Section 4.1. CD4, cluster of differentiation 4; CD8, cluster of differentiation 8; DCs, dendritic cells; IL, interleukin; IRAK-4, interleukin-1 receptor-associated kinase 4; IRF, interferon regulatory factor; JAK 1/2, Janus kinases 1/2; JNK, c-Jun N-terminal kinase; LCs, Langerhans cells; MHC I, class I major histocompatibility complex; NK cells, natural killer cells; PAMPs, pathogen-associated molecular pattern; PKR, double-stranded RNA protein kinase R; STAT-1, signal converter and transcription activator 1; TAP, transporter, associated with antigen processing; TFs, transcription factors; TGFβ, transforming growth factor β; TLR-9, toll-like receptor 9; TNFα, tumour necrosis factor α; TYK2, non-receptor tyrosine protein kinase 2.

The interferon response is one of the innate immune response pathways [104,122]. The HR HPV E6 and E7 proteins suppress many targets of the interferon response pathway. For example, E6 binds to the interferon regulatory factor (IRF) 3, while E7, on the other hand, binds to IRF-1 to block its functionality [119]. Interferon target genes such as STAT-1 are also suppressed by the HR HPV E6 and E7 proteins [123,124]. In this context, the E6 oncoprotein suppresses both the double-stranded RNA protein kinase R (PKR) via its co-localization with p-bodies [125] and the non-receptor tyrosine protein kinase TYK-2, the first member of the JAK family to be described, required for the activation of STAT-1 [119,126] (Figure 2A). E5, E6, and E7 oncoproteins influence Janus kinase/signal converter and activator of transcription (JAK/STAT) pathways to evade immune surveillance and thus maintain chronic infection. While STAT1 and STAT2 influence immune signaling via interferons, STAT3 and STAT5 are mainly associated with the proliferation and progression of many carcinomas. Morgan et al. were writing a good review article on this [127]. Moreover, the expression of the Toll-like receptor 9 (TLR9), known as the cluster of differentiation CD289, which recognises the pathogen-associated molecular patterns (PAMPs) (here, specifically: non-methylated CpG oligonucleotides (DNA)), which are characteristic for a wide range of microorganisms, is inhibited by the HR E6 and E7 oncoproteins [128] (Figure 2B). The DNA-activated antiviral innate immune response can also be inhibited by oncoproteins such as E7 from HPV and E1A from adenovirus. It was found that the cyclic guanosine monophosphate (GMP) adenosine monophosphate (AMP) synthase (cGAS) detects foreign DNA and signals it via the adapter protein, the stimulator of interferon genes (STING). This is carried out by the LXCXE motif of these oncoproteins. As a consequence, this prevents the phosphorylation of IRF3 via TANK-binding kinase 1 (TBK1). In this way, IRF3 cannot translocate to the nucleus to trigger the authorisation of inflammatory genes [129]. Other authors have also found that HPV-16 and other oncoviruses cause the methylation and acetylation of histones upstream of the TLR9 transcription starting point via the E7-mediated binding of the histone demethylase JARID1B and histone deacetylase HDAC1 to the inhibitor transcription complex, consisting of NF-κB, p50, p65, and oestrogen receptor alpha (ERα) and thus negatively regulate immunity via the TL9-mediated interferon reaction [130]. The virus’ “tactic” is not just to repress the targets of the interferon response; on top of that, it suppresses the low but constitutively expressed levels of interferon κ and its induced genes in keratinocytes [131].

An infection is usually associated with increased inflammatory cytokine production [132]. In HPV-infected keratinocytes, however, there is a reduced expression of inflammatory cytokines, such as interleukin 1 (IL-1), IL-6, tumour necrosis factor α (TNF-α), and transforming growth factor β (TGF-β) [133,134,135] and, in parallel, increased expression levels of anti-inflammatory cytokines such as IL-10 [128,133]. As a result, the infected host tissue is less infiltrated by activated CD8^+^ and CD4^+^ lymphocytes, Langerhans cells (LCs), and other dendritic cells (DCs) [104,136]. It is probably also possible that HR HPV L2 protein can negatively influence the maturation, migration, and cytokine secretion of LCs via the suppression of the E-cadherin level and that this leads to an attenuated crosstalk between LCs and keratinocytes [128,137] (Figure 2C).

Another component of the immune response is the class I major histocompatibility complex (MHC I), which controls the MHC pathway via the antigen presentation and is expressed in almost all cells on their surface. The HR HPV oncoproteins E5, E6, and E7 are responsible for down-regulating MHC I expression and probably cause the suppressed or delayed T cell reaction [138,139]. E7 down-regulates the antigen peptide transporter, associated with antigen processing (TAP) [128]. Finally, the functionality of the natural killer cells is also restricted [119,138] (Figure 2D). By suppressing the innate and adaptive immune response, human papillomaviruses have managed to create a persistence of the infection, which, however, also favours the malignant transformation in parallel.

### 4.2. Increased Angiogenesis Rate Due to Increased Oxygen and Nutrient Requirements within the HPV Lesions

**Figure 3:**
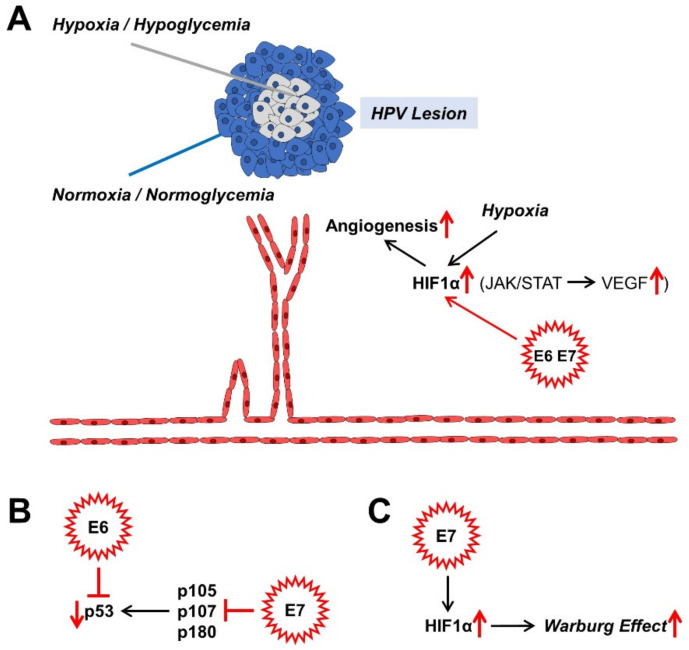
Effects of HPV oncoproteins on angiogenesis (**A**), malignant transformation (**B**), and metabolism (**C**). The mechanisms shown are mainly described in sections: 4.2. (**A**), 4.3. (**B**), and 4.4. (**C**). HIF1α, hypoxia-inducible factor 1α; JAK, Janus kinase; STAT, signal converter and transcription activator; VEGF, vascular endothelial growth factor.

If HPV infection persists, the benign or malignant lesions that it causes also grow over time. There is competition for nutrients and oxygen in the centre of the proliferating lesions. Normally, this leads to cell cycle arrest, which can lead to cell death. Angiogenesis, the formation of new blood vessels through sprouting and splitting reactions from existing blood vessels, counteracts cell death [70,140,141]. Moreover, the hypoxic nucleus of the lesion causes an up-regulation of hypoxia-inducible factor 1 (HIF1), a transcription factor; and the encoded genes associated with the hypoxia response element (HRE) for erythropoiesis, glycolysis, and angiogenesis are upregulated due to the oxygen deficiency [142]. These processes affect both benign and malignant lesions. It is both the high-risk proteins of E7 and those associated with a low risk of cancer progression that increase the level of HIF1α and its target genes via the epigenetic displacement of the histone deacetylases (HDACs) from the HIF1–histone complex [143,144,145,146,147,148]. Angiogenesis is, therefore, crucial for the further growth of the HPV lesions, which can now undergo malignant transformation (Figure 3A).

### 4.3. The Role of HR HPV Proteins in Malignant Transformation

In particular, the two oncoproteins E6 and E7 occur individually or in combination to ensure that the HPV infection is maintained in the cervix and other tissues. Only the high-risk types of E6 and E7 cause the transformation to malignant progression. The targets of these two oncogenic proteins originate from the various regulatory mechanisms of the cell, such as the cell cycle, gene expression, replication, and signalling. We want to discuss these in detail now.

An important building block in maintaining replication in differentiating cells of the TZ of the cervix is the binding to members of the retinoblastoma (pRb) protein family. These are considered tumour suppressors. They include p105 (pRb), p107 and p130 (Figure 3B). Normally, they repress E2F transcription factors (TFs) in order to prevent the entry into the S phase of the cell cycle by down-regulating the expression of S-phase-associated genes and thus, to cause cell cycle arrest. High-risk E6 and E7 proteins have a higher binding affinity for the TFs than their low-risk counterparts do. Thus, the suppression of these tumour suppressor proteins causes the cell cycle to progress, which is eminent for HPV persistence and malignant progression. In the case of HR HPV E7, even pRb degradation occurs [149]. In addition, other factors, such as the histone deacetylases 1, 2, and 3, which activate different genes, for instance the eukaryotic E2F2 transcription factor, bind to the E7 protein. E2F2, which is activated by EF2 and MYC proteins, ensures the maintenance of the virus genome in undifferentiated cells and its amplification in differentiated cells [150]. The cell cycle progression is promoted on the one hand by binding of E7 to the inhibitors of cyclin-dependent kinases p21 and p27, and on the other hand, by binding to cyclins A and E [149]. The member of the Polycomb complex E2F6 [151], ataxia telangiectasia, mutated (ATM) DNA damage sensor [152], and p600 (600 kDa protein) as a retinoblastoma-binding protein [153,154], bind to E7 and suppress the anchorage-independent cell death (anoikis). All these adaptations are due to the “efforts” of the virus to preserve itself from the “dead end” of the cell cycle arrest of differentiated keratinocytes within the cervix and to promote proliferation.

An accompanying reaction to the abrogation of the pRb tumour suppressor-functionality is the compensatory stabilisation of the tumour suppressor-protein p53, which negatively influences virus replication and can trigger apoptosis. In terms of developmental biology, the HR E6 oncoproteins have therefore evolved to prevent apoptosis via the degradation of p53 and the immortalisation of the keratinocytes [155] (Figure 3B). With their approximately 150 amino acids (AA) in length, HR E6 proteins are not enzymes and rather act as scaffolding proteins for other factors. P53 is rapidly broken down and inactivated via a trimeric complex of E6, the E3 ubiquitin ligase E6AP, and p53 itself [155]. In contrast, low-risk (LR) E6 proteins also bind to the ubiquitin ligase; however, they do not lead to the desired breakdown of p53. Therefore, there must be other factors, which drive p53 degradation. The reduced degree of acetylation of p53 via the binding of E6 to the histone acetyltransferases, the CREB1-binding protein (CBP) and p300 (CBP/p300) and hADA3, all of which are p53 coactivators, further inactivates p53. However, mutated E6 variants can also immortalise keratinocytes without inactivating p53, which suggests that E6 influences other functionalities of the cell [155]. One of these functions is the expression of the human telomerase reverse transcriptase (hTert), which, activated by HR E6 proteins, binds and activates a number of other factors such as the proto-oncogene c-Myc and its interaction partner, the transcription factor Max and its cooperation partner Sp1 [156], E6AP, and its target, the X-Box binding protein NFX1-91. In contrast to the HR E6 proteins, the low-risk (LR) E6 proteins do not result in any activation of these described immortalisation factors. In order to immortalise keratinocytes, it is therefore necessary for E6 to cooperate with E7 in order to activate hTert via E6 and deactivate pRb via E7 [155]. The PDZ domain of membrane-bound proteins enables E6 to bind to its C terminus; and so, it mediates the proliferation and maintenance of the HPV virus genome. Thus, through the cooperative influence exerted by the tandem of E6 with E7 on the cellular regulatory mechanisms of the HPV-infected cervix cell, its malignant progression is also promoted.

### 4.4. HR HPV Oncoproteins and Their Effects on the Altered Cell Metabolism

As long as the replication cycle of the virus is complete, and its DNA—the tandem of E6 and E7—does not integrate into the host genome, the HPV infection, and this also applies to the HR HPV variants, will be completely eliminated by 90% of cases from the host over the course of about 2 years, mediated by a potent immune system [157]. In this case, tumourigenesis does not occur either. If the infection of the cervical tissue with high-risk HPV variants lasts longer within the viral lesions, genomic instability of the host DNA and usually integration of the virus DNA occurs, probably due to the effects of HR E6 and E7 on its target genes. Moreover, this leads to the overregulation of the E6 and E7 DNA segments, to the accumulation of mutated DNA and to cancer development [52,53]. The damage to the E2 gene or the methylation of its binding sites in the locus control region (LCR) probably leads to a reduced repression of the E6/E7 expression level [52,158]. The integration of the E6/E7 tandem into the host genome sometimes leads to so-called “super-enhancer” regulatory elements, which intensify E6/E7 expression in a hyperactive manner [159]. The ratio of integrated HPV gene copies to episomal viral DNA increases as the lesion moves towards malignant progression [160]; and in the cervical cancer cells—due to the lack of viral episomes—no virus is synthesised anymore. Ultimately, paradoxically, the virus ends its own development with the onset of tumourigenesis.

The previously described cooperative repression of the tumour suppressor proteins pRb and p53 and the associated apoptosis suppression by HR HPV E6 and E7 leads, via the E6-mediated blockade of the p53-activated regulator of glycolysis and apoptosis TIGAR (TP53-induced glycolysis and apoptosis regulator), to the activation of glycolysis (Figure 1). In addition, via the cytochrome C oxidase-assembly protein (SCO2), it leads at the same time to the inhibition of oxidative phosphorylation (OxPhos) [161,162]. The high-risk HPV16 E6 protein not only regulates the glycolytic metabolic pathways via p53, which is also vital for cancer [72]. It also binds to the transcription factor c-Myc, responsible for up-regulating the expression level of a large number of rate-limiting enzymes in glycolysis, such as (α-)enolase 1 (ENO1), hexokinase 1/2 (HK1/2), lactate dehydrogenase A (LDHA), phosphofructokinase 1/2 (PFK1/2), and glucose transporters 1–4 (GLUT1–4) during aberrantly increased aerobic glycolysis (Warburg effect) [163,164]. The HR HPV16 E6 protein also influences the activity of the phosphoinositide-dependent kinase 1 (PDK1) and mammalian target of the rapamycin complex 2 (mTORC2) pathways via the PI3K/AKT (PKB) pathway through the activation of the mTORC1 signalling [165]. The latter serves the cell as a metabolic sensor which, regulated by growth factors, leads to the accumulation of the transcription factor HIF1, which is adapted to hypoxia and induced by it, in the nucleus and regulates proteins via the transcribed HRE gene region, such as, e.g., the up-regulation of the expression of glucose transporter 1 (GLUT1) [166]. HR HPV16 E6 also activates the pyruvate kinase isoform 2 (PKM2), which acts as a co-activator of HIF1 [166]. The influence of the hypoxic microenvironment by shifting the metabolic equilibrium from oxidative phosphorylation to anaerobic glycolysis leads to acidification of the cervical tissue. In addition to the stimulating effects on carcinogenesis via altered immune and metabolic signalling, this so-called lactate acidosis and the resulting lower pH value also have a beneficial effect on the infectivity of the virus, as the virus uses this stimulus from the lower pH value to penetrate the host cell via the endocytic pathways [167].

HR HPV16 E7 also seems to activate HIF1, at least in oral squamous cell carcinoma [168] (Figure 3C). HR HPV16 E5 indirectly activates the Warburg effect via EGF-stimulated epidermal growth factor receptor (EGFR) signalling and the subsequent activation of the extracellular signal-regulated kinases 1/2 (ERK1/2) and AKT [169,170]. In addition to the GLUT transporters, which transport glucose and other monosaccharides, polyols, and other small carbon compounds by means of facilitated diffusion, there are also sodium glucose transporter (SGLT) Na^+^/glucose co-transporters [171,172,173,174,175,176], which meet the high nutrient requirements of cancer cells. It provides a secondary active transport when glucose uptake by cancer cells is insufficient but requires energy in the form of adenosine triphosphate (ATP). For example, the E6 protein of HR HPV18 regulates the SGLT1 transporter [177]. EGFR is often strongly expressed and activated in cancer cells and co-associated with SGLT1 in the cell membrane. This stabilises the Na^+^/glucose co-transporter and increases the influx of glucose [171,172,173,178]. The over-expression of GLUT1 by HPV E6 via the repression of the tumour suppressor p53, which represses GLUT transporter expression, also leads to an increase in glucose uptake in cervical cancer [179]. HPV16 E6 is also able to hinder the von Hippel-Lindau (VHL)/HIF1α interaction and thus to increase GLUT1 expression and the Warburg effect by activating HIF1α in lung cancer cells [180,181]. HPV18 E6 co-expression in primary *Xenopus laevis* oocytes also led to a significantly increased distribution of SGLT1 in the cell membrane [177].

Overall, the HPV E6 and E7 oncoproteins intervene at various points in the glycolytic pathway, but also in the Krebs cycle. For example, by inactivating p53, as the antagonist of G6PDH, the key enzyme in the PPP, increases and, with it, the nucleotide levels. The HPV E6 oncoprotein also positively regulates pyruvate dehydrogenase kinase 2 (PDK2) and subsequently negatively regulates pyruvate dehydrogenase (PDH), thereby lowering the acetyl CoA level and shifting the metabolism towards lactate production, another hallmark of cancer. Furthermore, the p53 degradation causes a reduced glutaminolysis via the down-regulation of glutaminase 2 (GLS2), which, via the reduction in the α-ketoglutarate level, attenuates the function of the Krebs cycle, which is also one of the hallmarks of cancer. The enzymatic activity of the foetal form (M2) of pyruvate kinase (PK), called PKM2 or tumour-M2-PK, is more enzymatically active in glycolytic metabolism than the adult form PKM1 [72,182]. It was shown that the HPV16 E7 oncoprotein, through direct binding to PKM2, causes its dimerisation, hence, its activity in glycolysis and glutaminolysis; and thus also the nucleotide level increases and the proliferation of embryonic fibroblast cells of the mouse (NIH-3T3) promotes increased glucose and serine consumption and the elimination of metabolic crosstalk between glucose consumption and glutaminolysis. In both mouse (NIH-3T3) cell lines, the transformation increased glutaminolysis and the positive correlation between alanine and lactate production [183]. The change in PK isoforms from the tetrameric M1 to the dimeric tumour M2 isoform and the associated metabolic drift in cancer metabolism due to HR HPV E6 and E7 proteins also leads to increased tumour progression [92]. An important enzyme that is responsible for the shift towards lactate production and away from the complete oxidation of sugars by the Krebs cycle and the OxPhos is lactate dehydrogenase A (LDHA). Short, non-coding microRNAs (miRNAs) are involved in the regulation of the Warburg effect [93,95] and miRNA 34a (miR-34a) in particular is a tumour suppressor, as it is upregulated by p53 [94]. The HPV E6 protein causes p53 targeting to break it down and thus increases the activity of LDHA and thus lactate production via the down-regulation of miR-34a [96].

P53, which is degraded by the viral oncoprotein E6 via the proteasome, induces the protein expression of mitochondrial proteins, e.g., cytochrome C oxidase 2 (SCO2) [162], apoptosis-inducing factor (AIF) [184] and ferredoxin reductase (FDXR) [185], which are responsible for the integrity of the mitochondria and the functionality of the oxidative phosphorylation (OxPhos) [186]. The HPV E2 protein negatively regulates HR HPV E6 and E7 oncoproteins. This protein changes its location and migrates back and forth between the cytoplasm and the nucleus. In the cytoplasm, E2 promotes apoptosis [187]; and in the cell nucleus, it provokes mitotic DNA strand breaks and instability of the chromosomes. This could lead to the integration of the HPV genome into the host genome [188]. However, it also binds to the membrane of the mitochondria and there initiates a ROS release via the change in the crystal morphology of the mitochondrial proteins, which leads to the stabilization of HIF1 in the cell nucleus and is associated with an increase in glycolysis [189]. Complex III proteins mediate the ROS production of the mitochondria [190] and complex V, better known as ATP synthase (F_0_F_1_-ATPase), regulates crystal morphology [191]. The ROS release is thus modulated via the interaction with the E2 oncoprotein. The increased ROS change cell proliferation and cell differentiation through their character as a secondary messenger in the homeostasis of the cell. However, due to mitochondrial changes or aerobic glycolysis (Warburg effect), increased ROS levels can lead to oxidative stress (OS), which damages DNA, lipids or proteins [72]. Various molecules of antioxidant systems, such as reduced glutathione (GSH), superoxide dismutase 1, 2 and 3 (SOD1/2/3), catalase (CAT), peroxidases (Prxs), and thioredoxins (Trxs) are able to combat oxidative stress (OS) effectively [192]. The HPV16/18 E1 and E2 protein co-expressions lead, for example, to the down-regulation of the activities of GSH and SOD1/2 to damage the DNA via increased ROS [193].

## 5. Consequences of HPV Persistence and Therapeutic Outlook

Cervical cancer, but also other cancers caused by human papillomaviruses, show properties of immortalised, undifferentiated cells. For its development, however, the HPV virus absolutely needs differentiating cell types, such as those found in the transformation zone (TZ) between the endo- and ecto-cervix. For virus replication, it is imperative that the virus produce extrachromosomal episomes. In cancer cells, however, the virus genomes are integrated into the host genome in the form of the E6 and E7 DNA segments. This is probably another reason why cancer cells do not produce virions. The virus replication cycle ends with the incorporation of its DNA into the host DNA. It is in fact the case that nude mice, which are immortalised through the incorporation of E6 and E7 oncoproteins do not automatically show tumourigenicity. HPV infection is necessary for this, but in itself is not sufficient to initiate the malignant transformation. The prolonged passage during cell cultivation and a second oncogene such as *ras* lead to further DNA mutations that drive the transformation [194]. The E6 and E7 oncoproteins cause anomalies in the centrosome cycle and an aberrant chromosome separation and aneuploidy to cause genomic instability [66]. The promotion of cell cycle progression by E6 and E7 proteins via the inactivation of pRb and p53 promotes the accumulation of abnormalities in the chromosomes of the cervical cells [66]. In addition to the virus replication cycle, which is adapted to the differentiation cycle of the cervical TZ cells, the promotion of angiogenesis and the Warburg effect, as well as the mechanisms of immune evasion, virus persistence, and the promotion of chromosome instability are the basis for the tumourigenesis of cervical carcinoma, even if it ends in a developmental “dead end” for the virus in the long term.

Resistance to chemotherapy and radiation therapy is an obstacle that is difficult to overcome on the way to healing the patient. The hypoxic microenvironment of the tumour and the aberrant metabolism via aerobic glycolysis (Warburg effect) are mainly responsible for this [195,196]. In HPV-positive cervical cancer cells, the enzyme hexokinase 2 (HK2) is increased compared to HPV-negative tumour cells due to the Warburg effect; and its inhibition leads to re-sensitisation to radiation therapy [197]. The receptor tyrosine kinase (RTK), the human epidermal growth factor receptor 2 (HER2/neu, ERBB2) is over-expressed in HPV-positive cancer cells and associated with the over-expression of HIF1α, which is responsible for an increased Warburg effect [198]. The sole use of HER inhibitors such as *lapatinib* in therapy leads to treatment resistance in a model of HER2/HPV-positive SiHa cervical carcinoma cells; however, by the combinational treatment with glycolysis inhibitors such as 2-deoxy-D-glucose (2-DG), a glucose analogue that is not metabolised by the cells, resistance can be abolished [199,200].

Finally, we would like to recommend a few review articles to the reader, which report on the positive effects of the microbiota on patient health and the alleviation of side effects from chemo-, radiation-, and immunotherapy [5,6,7]. Three of these review articles are primarily about the intestinal microbiota and its positive effects on the side effects of chemotherapy and immunotherapy, such as: e.g., therapy with immune checkpoint inhibitors (ICIs), which target the receptor of the programmed cell death 1 ligand 1 (PD-1)/programmed cell death 1 ligand 1 (PD-L1) path and, in a larger number of patients, produce undesirable serious side effects. Here, for example, a faecal microbiota transplant (FMT) can improve the anti-tumour effect of PD-1 blockade in germ-free or antibiotic-treated mice. The intestinal bacterium *Akkermansia muciniphila* had a positive correlation with the clinical effectiveness of the therapy, which restored the effectiveness of the PD-1 blockade via interleukin-12 through the accumulation of CCR9^+^CXCR3^+^CD4^+^ T lymphocytes in the tumour beds [6].

Another publication [4] presents the positive effects of *Lactobacillus crispatus*, and the drug *Lactobacillus crispatus* CTV-05 (LACTIN-V) derived from it, on vaginal health; and the authors seek to restore women’s vaginal health by replenishing the dysbiotic vaginal microbiota (VMB) with this hydrogen peroxide (H_2_O_2_)-producing bacterium, thereby preventing recurrent inflammation and bacterial vaginosis (BV).

## 6. Conclusions

The influence of the vaginal microbiota and its interactions with the microbiota of other epithelial locations in women in diseases of the urogenital tract, including malignant transformations, has only recently been investigated in more detail. Clinical studies with a larger number of patients must follow, in order to recognise their value for a putative clinical treatment and to make valid statements about the effectiveness of probiotic treatment methods. Since there are also, probably, differences in the epidemiological development of cervical cancer between women of different origins, the ethnic and socio-economic origins of the patients must also be taken into account. In the future, questions about the age of sexual maturity, the number of children, the type of contraceptive, and possible influences from the effects of hormone preparations or the question about accompanying sexually transmitted diseases, triggered by pathological microorganisms should be raised. Interestingly, in women of Spanish origin, for example, the occurrence of cervical cancer was associated with the increased incidence of the pathological species *Sneathia* and the reduction in the number of symbiotic species of *Lactobacillus*, as well as an increase in the pH value of the vagina. Nevertheless, the knowledge about the mechanisms of the crosstalk between host and microorganism is still limited and needs further clarification. The influence of the microbiota on tissue homeostasis and the positive effects of a healthy probiotic epithelial landscape certainly deserve our special attention, especially in the prophylaxis of urogenital diseases, in order to maintain and promote the quality of life and health of women. In order to get back to our provocative title, and to the question of which of the many microorganisms is good and which is bad, one must surely come to the conclusion that this depends on the respective species, the tissue context and the respective pathological situation. The answer to our question is therefore in the grey area and should still take some effort if it can ever be answered.

## Data Availability

Not applicable.
